# *Acinetobacter* spp. porin Omp33-36: Classification and transcriptional response to carbapenems and host cells

**DOI:** 10.1371/journal.pone.0201608

**Published:** 2018-08-02

**Authors:** Katarina Novović, Sanja Mihajlović, Miroslav Dinić, Milka Malešević, Marija Miljković, Milan Kojić, Branko Jovčić

**Affiliations:** 1 Institute of Molecular Genetics and Genetic Engineering, University of Belgrade, Belgrade, Serbia; 2 Faculty of Biology, University of Belgrade, Belgrade, Serbia; Zhejiang University, CHINA

## Abstract

*Acinetobacter baumannii* has been recognized as one of the most challeging pathogens in clinical settings worldwide. Outer membrane porins play a significant role in *Acinetobacter* antibiotic resistance and virulence. *A*. *baumannii* carbapenem resistance and virulence factor porin Omp33-36 was the subject of this study. We investigated the *omp33-36* gene transcriptional response in the growth phase, its response to carbapenems, and the effect of contact with host cells. Additionally, the cytotoxic effect of *A*. *baumannii* towards keratinocytes was assessed, as well as correlation between *omp33-36* gene transcription and cytotoxicity. Further, *Acinetobacter* spp. Omp33-36 was classified and its characteristics relevant for vaccine candidature were determined. The level of the *omp33-36* gene transcription varied between growth phases, but a common pattern could not be established among different strains. Treatment with subinhibitory concentrations of carbapenems decreased, while contact with keratinocytes increased *omp33-36* expression in the analysed *A*. *baumannii* strains. Variations in *omp33-36* mRNA levels did not correlate with cytotoxicity levels. Decrease of *omp33-36* mRNA during treatment with subinhibitory concentrations of carbapenems, indicated the importance of transcriptional changes in reversible resistance to carbapenems due to the absence of Omp33-36. The transcription of *omp33-36* increased after contact with keratinocytes, indicating the important role of *de novo* transcription during the initial phase of *A*. *baumannii* infection. Primary structural analysis of *Acinetobacter* spp. Omp33-36 revealed three distinct groups (among four *A*. *baumannii* variants). Although we have shown that Omp33-36 was highly polymorphic, we propose a potential antigen (PLAEAAFL motif) for vaccine development. According to PROVEAN analysis, the highly polymorphic structure of Omp33-36 porin should not influence its function significantly.

## Introduction

*Acinetobacter* spp. are causative agents of infections that are predominantly nosocomial in origin, although community-acquired infections are increasingly reported as well [1]. Although species of the genus *Acinetobacter* are predominantly non-pathogenic, pathogenic members have been recognized as one of the most challenging in clinical settings. *Acinetobacter baumannii* causes the vast majority of *Acinetobacter* infections, followed by *Acinetobacter pittii* and *Acinetobacter nosocomialis* [2].

The pathogenic success of *Acinetobacter* species has been enabled by intrinsic resistance to a number of antibiotics, disinfectants and desiccation, as well as by its ability to easily acquire antibiotic resistance and virulence via horizontal gene transfer [1]. The increasing resistance to last resort antibiotics effective against *Acinetobacter*, such as carbapenems, has significantly limited therapeutic options [3]. Furthermore, reported pandrug-resistant *A*. *baumannii* strains make the situation more alarming [4]. Due to these findings, the World Health Organization (WHO) has positioned carbapenem-resistant *A*. *baumannii* on the top of the list compiled of pathogens for which new antibiotics are urgently needed [5]. In addition to new antibiotics, active and passive immunization, among other approaches, have been proposed for eradication of *Acinetobacter* infections [1].

The porins involved in the carbapenem resistance of *A*. *baumannii* are carbapenem resistance-associated outer membrane protein (CarO) and Omp33-36 [6,7]. CarO porin forms a channel with a binding site specific for imipenem, while specific binding sites for carbapenems in Omp33-36 porin have not been described so far [7]. It has been established that Omp33-36 porin participates in *A*. *baumannii* virulence and fitness [8]. The Omp33-36 porin facilitates adherence, invasion and cytotoxicity of *A*. *baumannii*, enabling survival in different organs and progression of infection within the host [8]. Additionally, Omp33-36 ability to modulate autophagy of human cells in order to survive within the host represents another aspect of its virulence [9]. Recently, several studies have assessed various outer membrane-associated vaccine candidates against *A*. *baumannii*, with promising results [10–16]. According to these studies, the Omp33-36 porin could be taken into account as a convenient target candidate for a preventive or therapeutic vaccine effective against *Acinetobacter* infections. It has been shown that cell density, treatment with subinhibitory concentrations of antibiotics and interaction with eukaryotic cells affect *A*. *baumannii* pathogenicity [17–21]. Although of significance, the role of Omp33-36 in those processes was not sufficiently studied.

In our previous study we revised the CarO porin classification of genus *Acinetobacter* [22]. Accordingly, the aim of this study was to investigate the prevalence and conservation of Omp33-36 porin among the genus *Acinetobacter* in order to establish whether it could be considered a good candidate for vaccine development. Furthermore, the dependence of *omp33-36* transcription on cell density, the effects of carbapenem treatment, and contact with keratinocytes were studied in carbapenem-susceptible and carbapenem-resistant *A*. *baumannii* in order to assess the contribution of Omp33-36 to the overall pathogenic potential of *A*. *baumannii*.

## Materials and methods

### Bacterial strains and species identification

One carbapenem-susceptible (813) and two carbapenem-resistant *A*. *baumannii* clinical strains (1995/12 –resistance due to oxacillinases production and 4031 –resistance due to oxacillinase production and inactivated *carO* gene) were isolated at the Institute for Mother and Child Health Care "Dr. Vukan Čupić", a tertiary care paediatric hospital in Belgrade, Serbia. The carbapenem-resistant strains were identified by a Vitek 2 automated system (BioMérieux, Marcy l’Étoile, France) and amplification of genes for 16S rRNA [23] in our previous study [22], while identification of carbapenem-susceptible strain 813 was done in this study by the same methods.

### The *carO* and the *omp33-36* gene analysis

The amplification and analysis of the *carO* and *omp33-36* genes were carried out as previously described [22,24]. The PCR products were purified with a GeneJET PCR Purification Kit (Thermo Scientific, Lithuania) and sequenced by the Macrogen service (Macrogen Inc., Netherlands).

### Carbapenem susceptibility testing

Carbapenem susceptibility of the strains used in this study was analyzed by the microdilution method, according to the European Committee on Antimicrobial Susceptibility Testing recommendations (http://www.eucast.org) [25].

### Subinhibitory carbapenem treatment

Bacterial cells were grown in Mueller-Hinton (MH) broth at 37°C with shaking overnight. Strains 813 and 1995/12 were grown in the presence of a subinhibitory concentration (1/10 of minimal inhibitory concentration—MIC- value) of meropenem or imipenem, while strain 4031 was incubated only with 1/10 MIC of imipenem. The selection of antibiotics was based on the role of CarO porin in imipenem resistance [7]. The bacterial cell density at 600nm (OD_600_) was monitored and stopped in different growth phases (early, middle, late exponential and stationary phase). The collected cells were centrifuged at 4500 *x g* for 5 min and used for RNA isolation. As controls, bacterial cells were cultivated in the absence of carbapenems. Experiments were done in triplicate.

### HaCaT keratinocyte infection with *A*. *baumannii*

A HaCaT keratinocyte cell line was cultured in high glucose DMEM supplemented with 2mM L-glutamine, 10% fetal bovine serum (FBS), 100U/ml penicillin and 100μg/ml streptomycin (Gibco, Life Technologies). For treatment, 1x10^6^ of HaCaT cells were plated in T25 cell culture flasks (Sarstedt, Germany) and incubated at 37°C with 5% CO_2_ overnight. Before infection the cells were washed in phosphate-buffered saline (PBS) and suspended in the aforementioned medium without antibiotics.

Bacterial cultures were grown in MH broth at 37°C with shaking overnight. The cells (1x10^8^ CFU/ml) were harvested and washed in PBS. After centrifugation at 4500 *x g* for 5 min, the pellets were suspended in high glucose DMEM containing 2mM L-glutamine and 10% FBS.

Infection of HaCaT keratinocytes with *A*. *baumannii* was performed by addition of the bacterial cell suspension to a flask with HaCaT cells (multiplicity of infection was 100). The HaCaT and *A*. *baumannii* cells were coincubated at 37°C with 5% CO_2_ for 21h. After treatment, the cell culture medium with bacterial cells was centrifuged at 405 *x g* for 5 min to pellet residual non-adherent HaCaT cells. For harvesting bacterial cells, supernatant was centrifuged at 4500 *x g* for 5 min and used for RNA isolation. Controls were incubated and purified as described above, but in the absence of HaCaT cells. Experiments were done in triplicate.

### Transcriptional analysis by real-time quantitative PCR (RT-qPCR)

The total RNA from *A*. *baumannii* cells was isolated with an RNeasy Mini Kit (Qiagen, Germany), with a modified lysis step [26]. DNase I treatment was performed by an Ambion DNA-free^™^ Kit (Thermo Fisher Scientific, MA, USA). Reverse transcription was done with a RevertAid RT Reverse Transcription Kit (Thermo Fisher Scientific, MA, USA) according to the manufacturer′s protocol. RT-qPCR was used for determination of *omp33-36* expression level. Primers and conditions applied for RT-qPCR are listed in Table 1 [27]. RT-qPCR was performed with a KAPA SYBR Fast qPCR Kit (KAPA Biosystems, MA, USA) in a 7500 Real Time PCR System thermocycler (Applied Biosystems, Thermo Fischer Scientific, MA, USA). Normalization was done against the *rpoB* gene using the ΔΔ*C*_*T*_ method (relative) [28]. The obtained values were then normalized against those from the early exponential phase in the absence of carbapenem. Also, the results from each growth phase were compared to those reached in the same phase without carbapenem treatment. Additionally, dependence of *omp33-36* transcription on contact with HaCaT cells was determined by normalization against values acquired from incubation without HaCaT cells. RT-qPCR experiments were done in triplicate.

**Table 1:** List of primers and conditions used in RT-qPCR.

Primer name	Sequence (5'-3')	Cycling condition	Reference
Omp33-RT_F	GCTTATCAATTTGAAGTTCAAGGTC	95°C/3 min	This study
Omp33-RT_R	GCTTGGTTTAAGAAAGCTGC	95°C/15 s-	
RpoB_For	TCCGCACGTAAAGTAGGAAC	60°C/60s	[27]
RpoB_Rev	ATGCCGCCTGAAAAAGTAAC	[40 cycles]	

### Cytotoxicity Assay

The cytotoxicity levels after infection of HaCaT keratinocytes with *A*. *baumannii* cells were measured with a lactate dehydrogenase (LDH) Cytotoxicity Assay Kit (Thermo Scientific, Lithuania). Quantitative determination of LDH released from dead cells was measured in the collected supernatants according to the manufacture′s protocol. The absorbance was measured at 490 nm in a microplate reader (Tecan Austria, Gmbh, Austria). Experiments were done in triplicate.

### Database search and data analysis

To build the Omp33-36 protein profile the PSI-BLAST [29] homology searches were restricted to either *Moraxellaceae* or *A*. *baumannii* with an e-value cutoff of 1e-4. All hits that exhibited at least 27% identity and over 71% of the query fragment length were retained for further analysis. As an Omp33-36 prototype, Omp33-36 CAH40841.2 was used. Proteins with sequence sizes significantly different from the average were removed. Amino acid sequences of Omp33-36 from the NCBI database (S1 File) and sequences from Serbian strains were aligned using Clustal W version 1.7 [30].

### Phylogenetic analysis

Phylogenetic trees were constructed using MEGA version 7.0 [31]. The Clustal W multiple sequence alignments were performed using default parameters. The maximum-likelihood (ML) method, using a Jones-Taylor-Thornton (JTT) distance matrix, was used for the construction of an Omp33-36 porin phylogenetic tree. The *A*. *baumannii* strains from this study were also involved in analysis. A bootstrap consensus tree inferred from 1000 replicates was taken to deduce confidence levels for the ML trees. An online tool (iTOL, v3) for the display and annotation of the tree was used [32].

### Topology prediction and analysis of Omp33-36 porin isoforms

Four *A*. *baumannii* Omp33-36 porin variants were included as representatives of *Acinetobacter* spp. Omp33-36 groups. Transmembrane topology predictions for the Omp33-36 isoforms were performed by PRED-TMBB (http://bioinformatics.biol.uoa.gr/PRED-TMBB) [33].

The Shannon entropy [34] of each amino acid position in Omp33-36, multiple alignments of the three main Omp33-36 groups among *Moraxellaceae*, was calculated using the following equation:

$$H=-\sum_{i=1}^{M}Pi{\text{log}}_{2}Pi$$

where the *Pi* is the frequency of amino acid residue *i* in that site and *M* is the number of amino acid types. Shannon entropy (*H*) ranges from 0 (only one residue is represented in a given position) to 4.32 (all 20 residues are equally represented in a given position). The amino acid changes observed in the different Omp33-36 variants relative to the canonical Omp33-36 variant 1 were analyzed using PROVEAN software (http://provean.jcvi.org/), which predicts whether an amino acid substitution, insertion or deletion has any impact on the biological function of a protein.

### Statistical analysis

All results are presented as mean values ± standard deviations. One-way ANOVA, followed by Tukey’s *post hoc* test, was used to compare multiple treatments. In experiments with HaCaT cells, a Student′s *t*-test was performed to compare differences between the control and experimental groups. Values at *p*0.05 or less were considered to be statistically significant. All data were analyzed using SPSS 20.0 v.

## Results

The strains used in this study were identified as *A*. *baumannii*. The selection of strains was based on carbapenem resistance phenotype.

### The *carO* and *omp33-36* gene analyses

The nucleotide alignment of the *carO* gene (S2 File) in carbapenem-susceptible strain 813, compared to the BLAST database, revealed 99% identity to the corresponding gene of the clinical strain *A*. *baumannii* C71 from China (KX517489.1), while the nucleotide sequence of *omp33-36* was 100% identical to an outer membrane protein gene of clinical strain *A*. *baumannii* IOMTU433 from Nepal (AP014649.1, from 219 644 to 218 747 bp). The *carO* and *omp33-36* nucleotide sequences of carbapenem-resistant strains 1995/12 and 4031 were determined in our previous study [22]. Nucleotide sequences of *omp33-36* genes from strains used in this study are reported in S3 File.

### Carbapenem susceptibility

The carbapenem susceptibility testing was performed for all strains, with the exception of 4031, which was not tested against meropenem due to its lack of functional CarO porin (Table 2).

**Table 2:** MIC values determined by microdilution method.

Strain	Imipenem MIC (μg/ml)	Meropenem MIC (μg/ml)
813	0.1 (S)	0.1 (S)
1995/12	16 (R)	32 (R)
4031	32 (R)	NT

S-susceptible; R-resistant; NT-not tested.

### Effects of growth phase and carbapenems on *omp33-36* gene transcription

**Fig 1:**
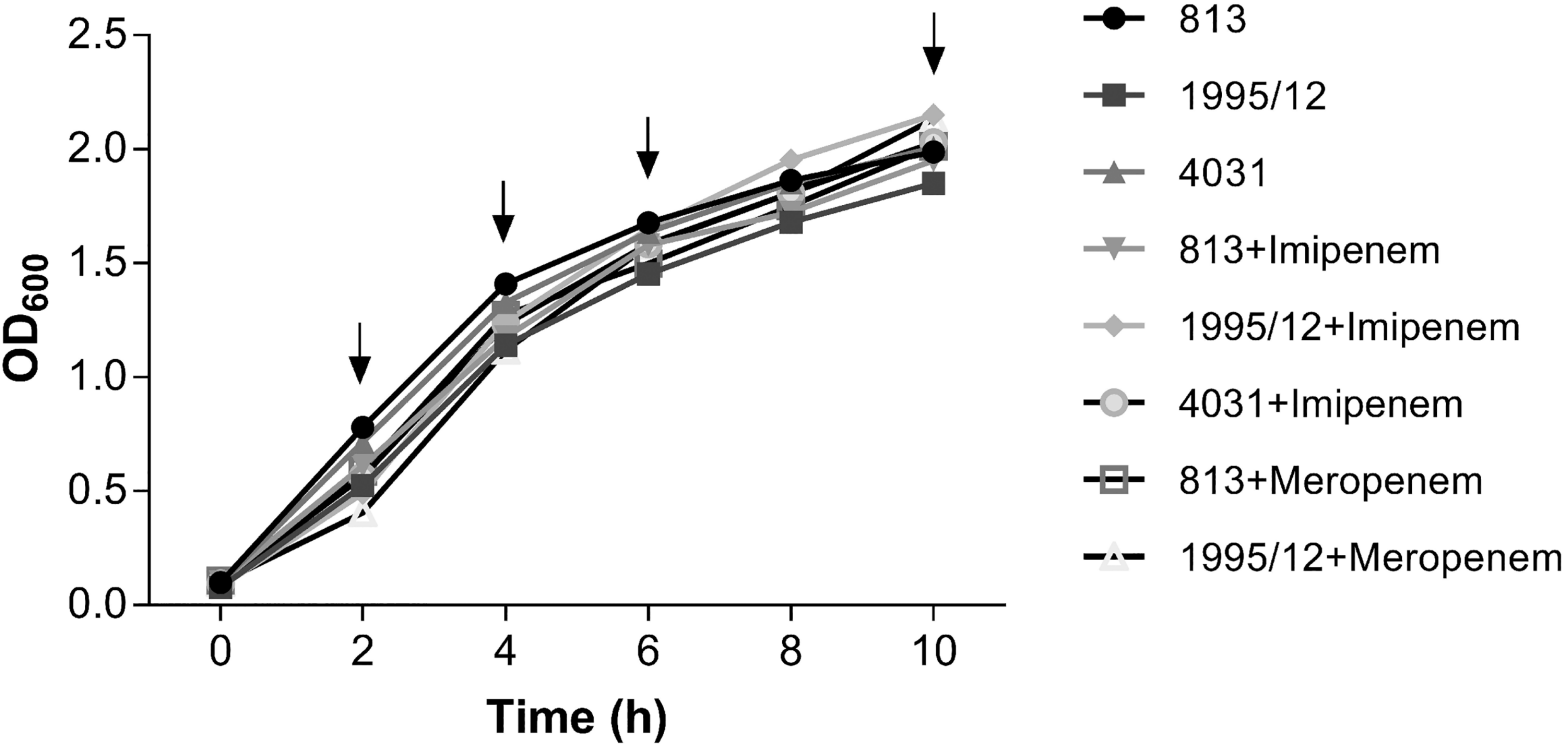
The growth curves of *A*. *baumannii* strains, untreated or treated with subinhibitory carbapenems (1/10 of MIC value). *A*. *baumannii* strains were incubated in the presence of a subinhibitory concentration of imipenem (813, 1995/12 and 4031) or meropenem (813 and 1995/12). The arrows indicate time points (2h, 4h, 6h and 10h) in which aliquots were collected for expression analysis.

**Fig 2:**
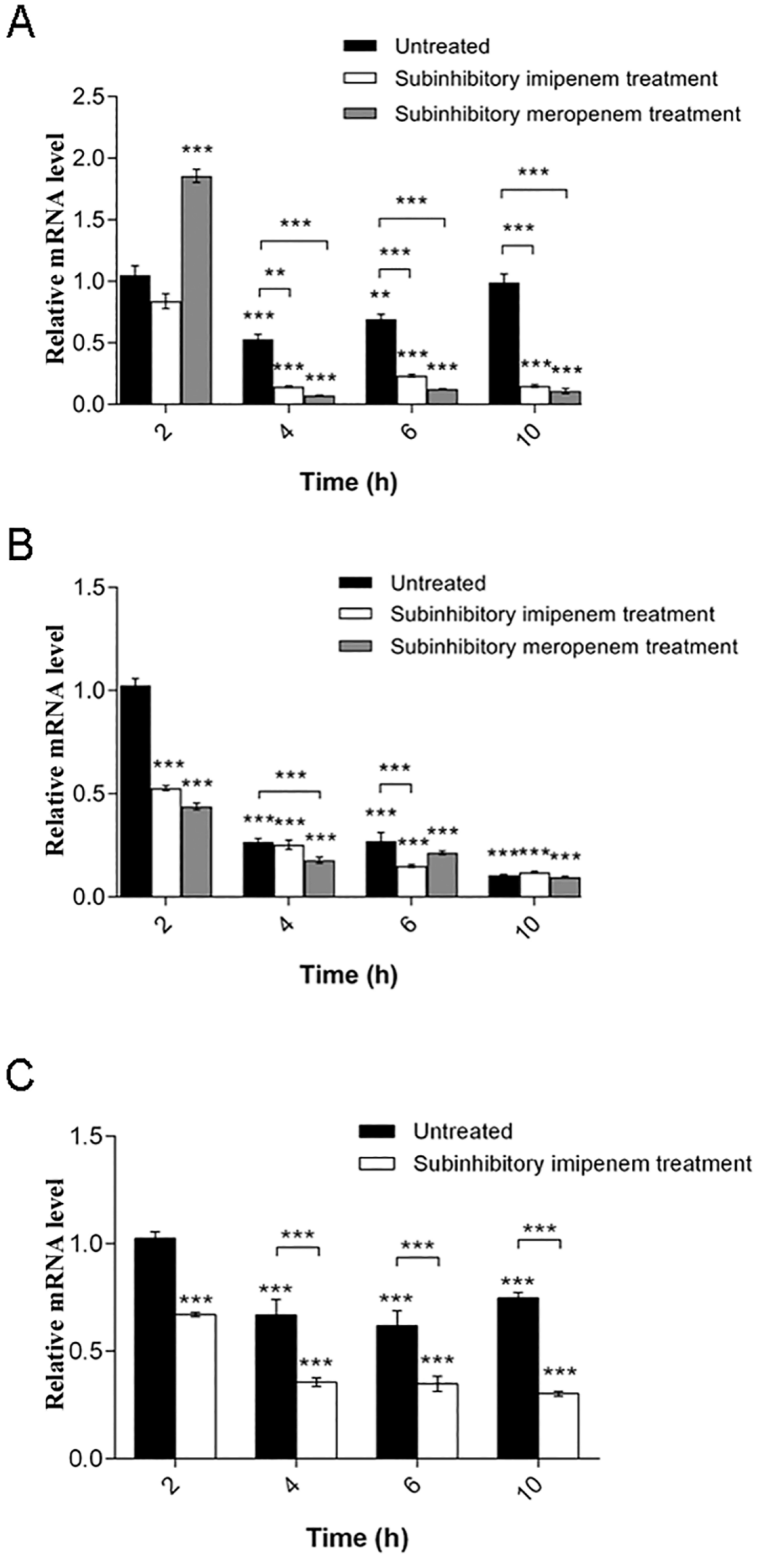
Changes of *omp33-36* mRNA levels during exposure to carbapenems through growth phases. (A) Carbapenem-susceptible strain 813. (B) Carbapenem-resistant strain 1995/12. (C) Carbapenem-resistant strain 4031. All expression results were normalized relative to *rpoB* by the 2^−ΔΔCt^ method. Values are the means from results obtained in triplicate. Error bars represent the standard deviation of the mean value. One-way ANOVA with Tukey′s *post hoc* test was used to compare the gene expression results relative to the expression of the *omp33-36* gene in the early exponential phase of an untreated strain and to the expression of the untreated strain for each time point (** *p*< 0.01, *** *p*< 0.001).

The growth curves of carbapenem-treated and untreated strains are presented in Fig 1. Time points corresponding to early (2h), middle (4h), late exponential (6h) and stationary growth phase (10h) were chosen according to measured OD_600_ values. Changes in the *omp33-36* mRNA levels during growth phases and exposure to carbapenems, investigated by RT-qPCR, are presented in Fig 2.

According to the obtained results, transcriptional expression of the *omp33-36* gene varied during different growth phases. All strains have shown a trend of *omp33-36* mRNA level decrease in transition from early to middle exponential phase (Fig 2). After this decrease, strains 813 and 4031 slowly returned to the *omp33-36* mRNA levels starting point (Fig 2A and 2C). In contrast, *omp33-36* mRNA levels showed a constant decline in strain 1995/12 (Fig 2B).

Subinhibitory concentrations of carbapenems reduced *omp33-36* gene transcription throughout growth phases in all strains (Fig 2). The change was most expressive in carbapenem-susceptible strain 813 (Fig 2A). The exception was meropenem treatment in the early exponential phase compared to the untreated control (Fig 2A).

### Effect of contact with keratinocytes on the *omp33-36* gene transcription

**Fig 3:**
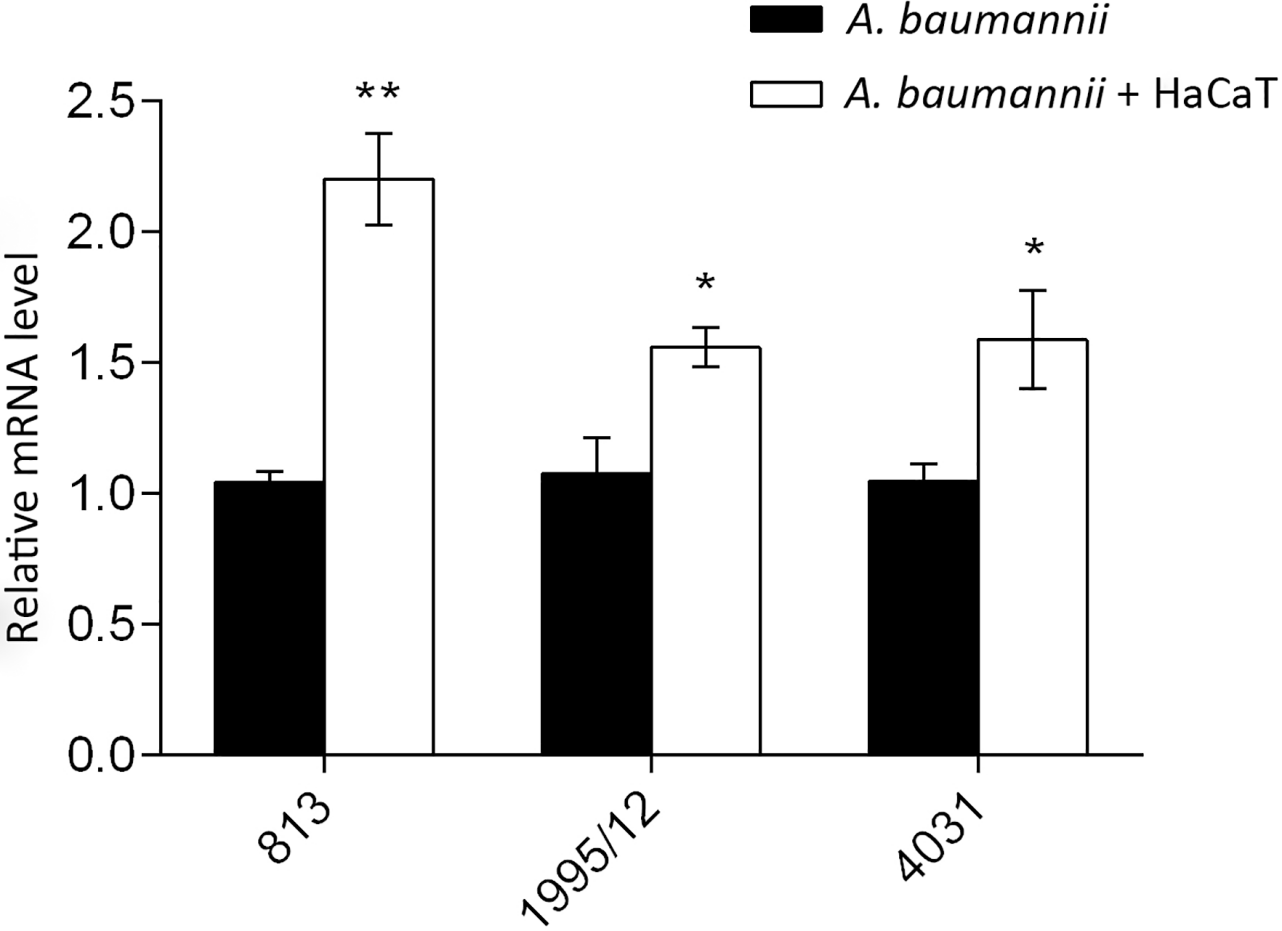
Inducibility of the *omp33-36* gene expression after contact with HaCaT keratinocytes. All expression results were normalized relative to *rpoB* by the 2^−ΔΔCt^ method. Values are the means of results obtained in triplicate. Error bars represent the standard deviation of the mean value. A Student’s *t*-test was used to compare the experimental group to the control (* *p*< 0.05, ** *p*< 0.01).

The impact of contact with HaCaT keratinocytes to the*omp33-36* gene transcription was investigated by RT-qPCR. All strains significantly increased their *omp33-36* mRNA levels after contact with HaCaT keratinocytes (Fig 3). The change was themost prominent in carbapenem-susceptible strain 813, while moderate in carbapenem-resistant strains 1995/12 and 4031 (Fig 3).

### Cytotoxic effect of *A*. *baumannii* strains on HaCaT keratinocytes

**Fig 4:**
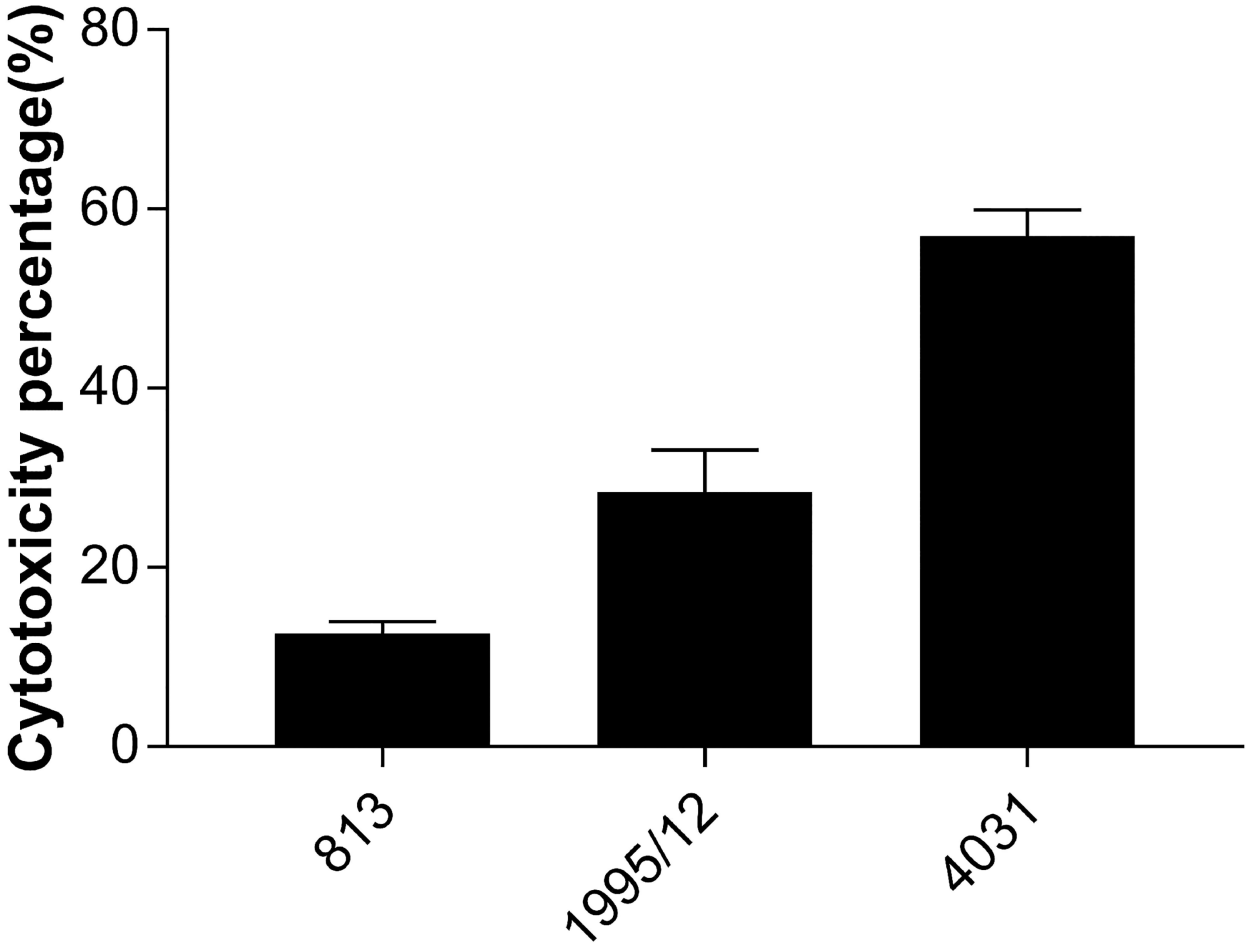
Cytotoxic effect of carbapenem-susceptible and carbapenem-resistant *A*. *baumannii* strains on HaCaT keratinocyte cells. Cytotoxic levels caused by carbapenem-susceptible strain 813 and carbapenem-resistant strains 1995/12 and 4031 on HaCaT cells were measured by LDH assay. Values are the means from results obtained in triplicate. Error bars represent the standard deviation of the mean value.

The results obtained from an LDH assay demonstrated that the most prominent cytotoxic potential was observed for carbapenem-resistant strain 4031 (56,97 ± 2,9%) followed by carbapenem-resistant strain 1995/12 (28,41 ± 4,7%). Carbapenem-susceptible strain 813 showed the weakest cytotoxicity on HaCaT cells (12,6 ± 1,34%) (Fig 4).

### Phylogenetic analysis and polymorphism determination of Omp33-36 porin

**Fig 5:**
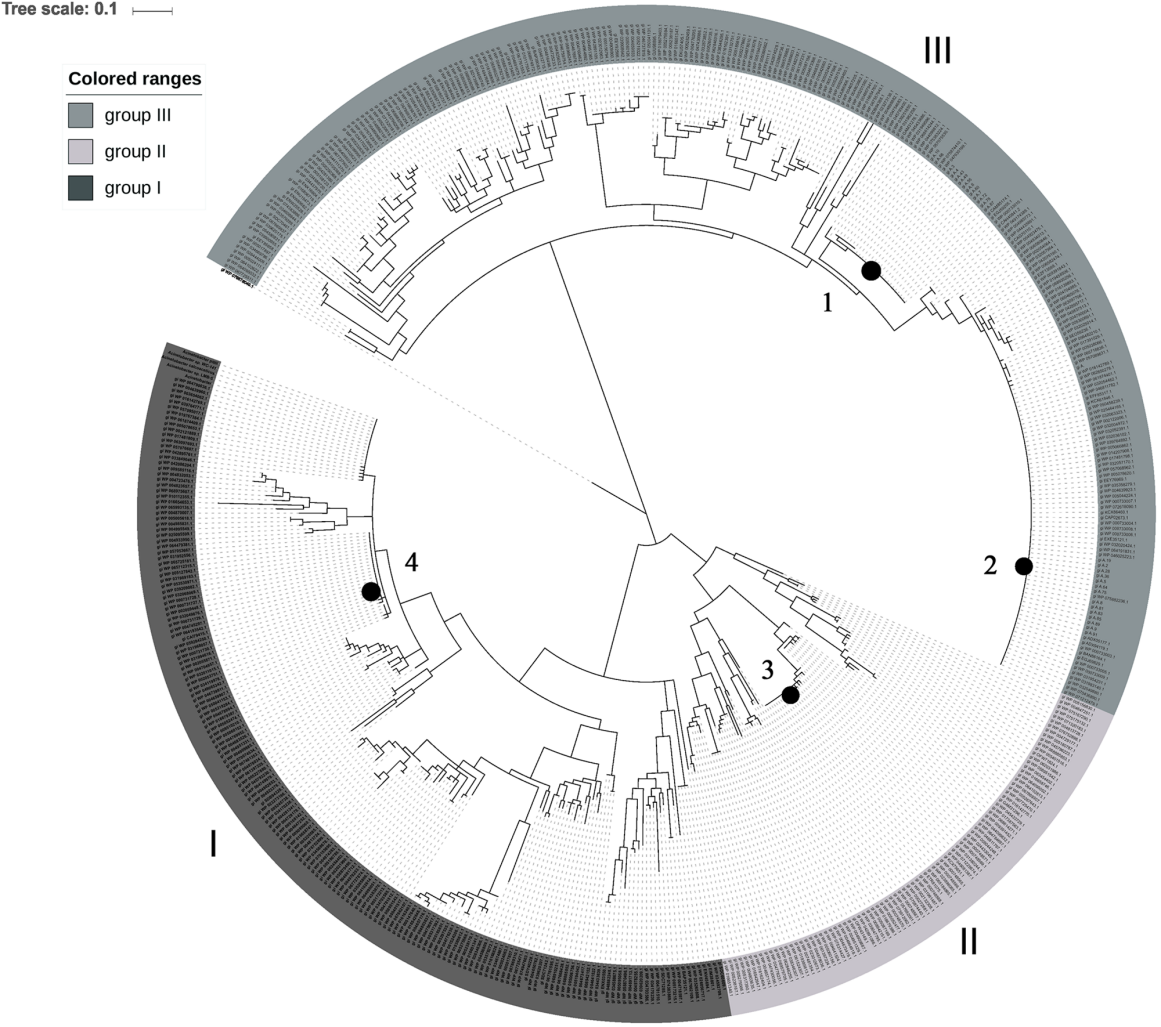
Phylogenetic inferences of Omp33-36 porin among *Acinetobacter* spp. A phylogenetic tree of Omp33-36 porins was constructed with the maximum likelihood (ML) method using a Jones-Taylor-Thornton (JTT) model distance matrix. The confidence levels were calculated from 1000 bootstrap resamples of alignment used for phylogenetic inferences by ML method. The gray shades represent the three dinstinct groups (I-III) consisting of *Acinetobacter* spp. from the database. *A*. *baumannii* variants are indicated by black circles. In group I is variant 4 in group II is variant 3 and in group III are variants 1 and 2.

**Fig 6:**
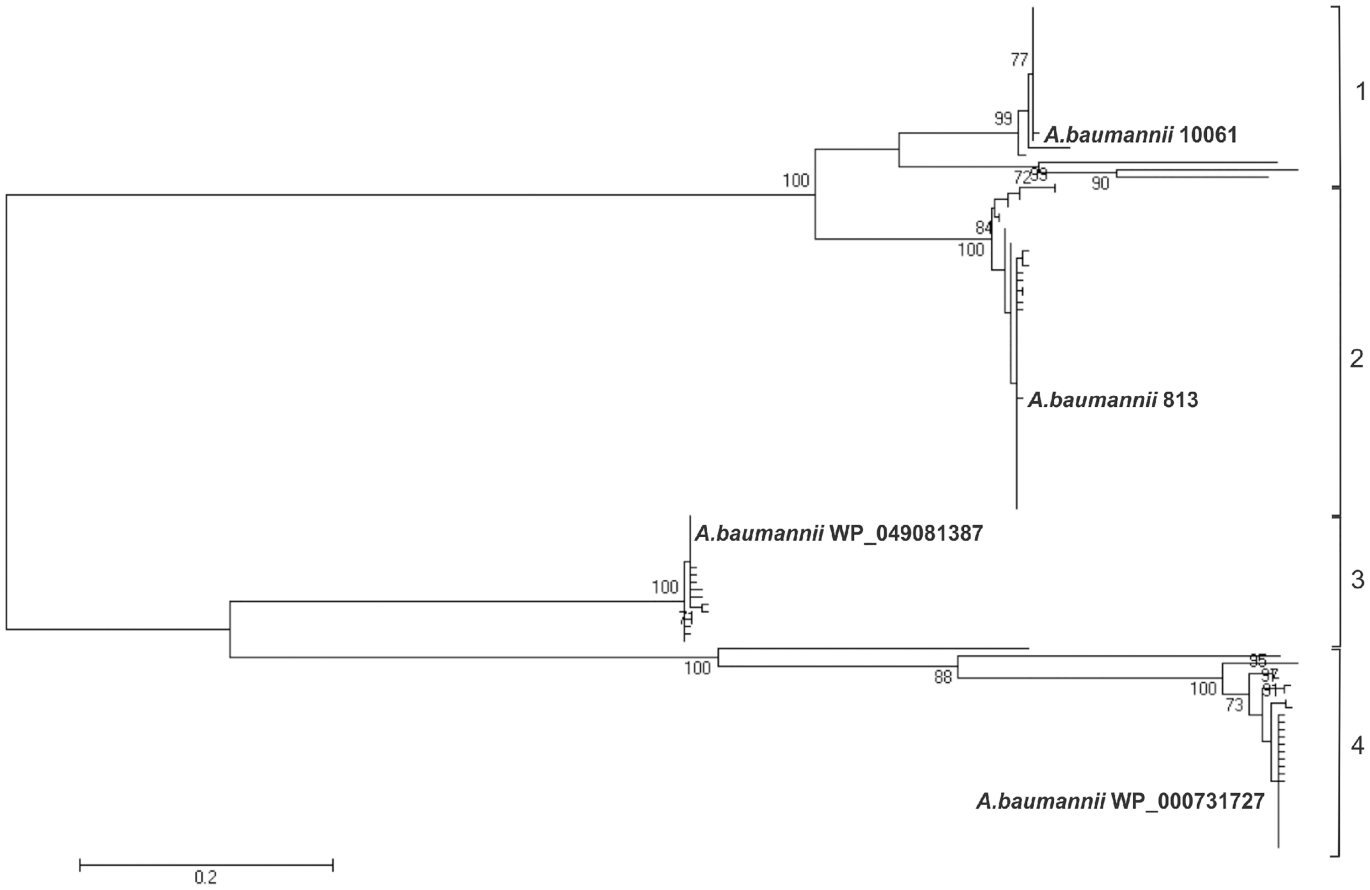
Phylogenetic inferences of Omp33-36 porins among *A*. *baumannii*. An ML phylogenetic tree was constructed from the Omp33-36 amino acid sequences available in the NCBI database. The corresponding Omp33-36 variant type (1–4) is indicated and given in bold. The representatives of variants 1–4 were 10061 (Omp33-36 from a Serbian *A*. *baumannii* strain [22]), 813 (Omp33-36 from Serbian *A*. *baumannii* strain used in this study), WP 049081387 (a putative *A*. *baumannii* porin from the NCBI database) and WP_000731727 (a putative *A*. *baumannii* porin from the NCBI database), respectively. The percentage of 1000 bootstrap resamples supporting the different clusters are indicated at the bifurcations (only bootstrap results above 70% are reported).

**Fig 7:**
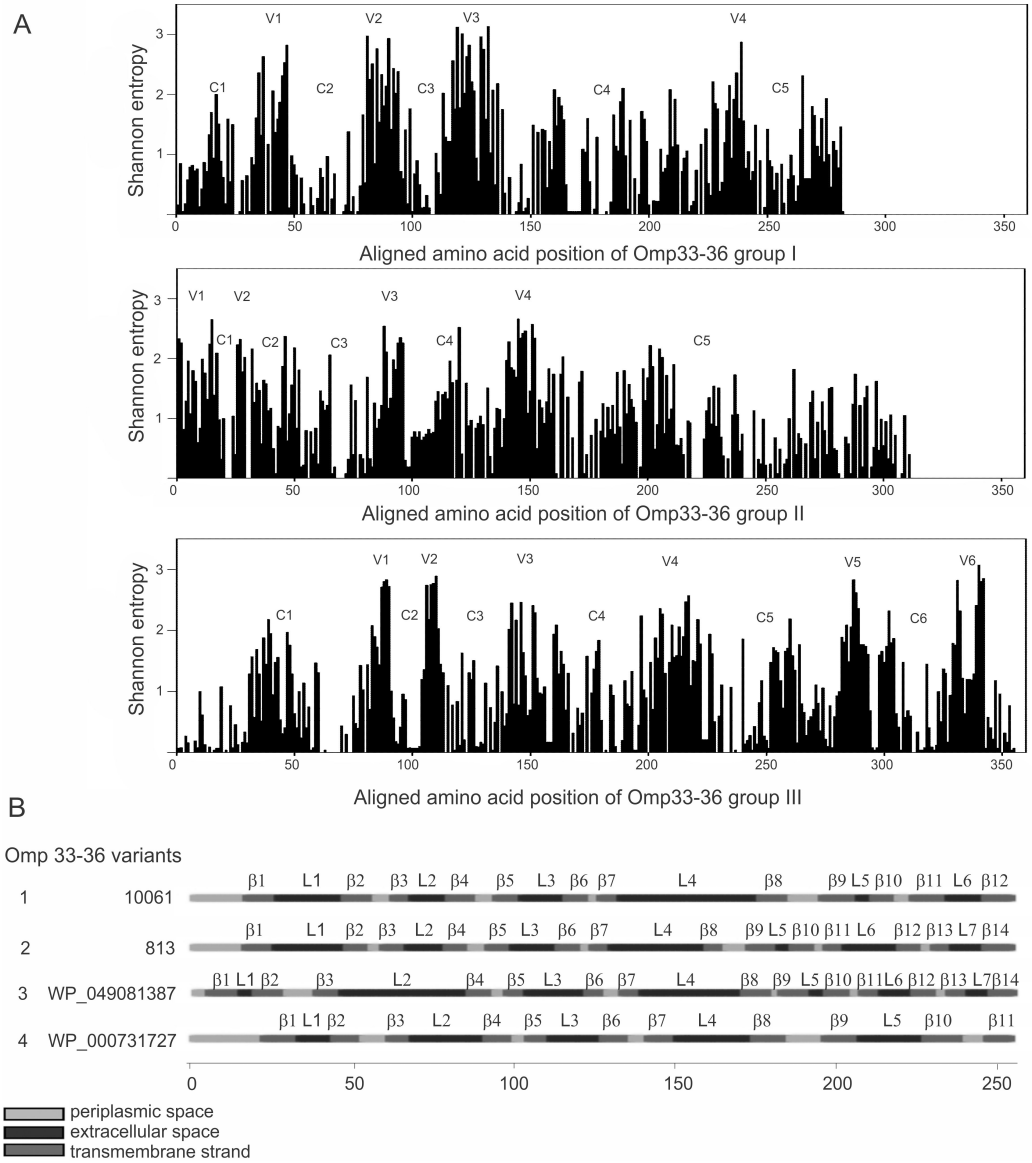
Distinct structural Omp33-36 porins among the *Acinetobacter* spp. population. (A) Shannon variability of Omp33-36 sequences within groups I, II and III. The height of each bar indicates the entropy of an amino acid residue at a given position. The conserved (C1-C6) regions and variable (V1-V6) regions are shown. (B) Predicted transmembrane topology of the different *A*. *baumannii* Omp33-36 variants (1–4) representing Omp33-36- groups. External loops (L1-L7), transmembrane spanning regions (β1-β14) and periplasmic regions are depicted.

The maximum likelihood (ML) phylogenetic analysis of Omp33-36 porins from the *Acinetobacter* genus separated Omp33-36 into three groups (I-III) (Fig 5). Furthermore, ML analysis of only *A*. *baumannii* species separated Omp33-36 into four clearly distinct isoform groups, referred to as *A*. *baumannii* Omp33-36 variants 1 to 4 (Fig 6). The most comprehensive, but least conserved, group III, was divided into two *A*. *baumannii* variants (1 and 2) (Fig 5). The Omp33-36 porin of carbapenem-susceptible strain 813 and carbapenem-resistant strain 1995/12 was classified as variant 2, while the Omp33-36 of carbapenem-resistant strain 4031 belonged to variant 1. The smallest, group II, includes Omp33-36 variant 3, while group I includes variant 4 (Fig 5). Multiple sequence alignment comparisons revealed that 86% of the amino acid positions of Omp33-36 porins were polymorphic. The polymorphic regions, as well as conserved regions, were well defined in all three Omp33-36 groups (Fig 7A). There were 87% polymorphic amino acid positions of Omp33-36 in group I (245 out of 284 amino acid positions), 83% in group II (260 out of 311 amino acid positions), and 87% in group III (310 out of 355 amino acid positions). Omp33-36 group I and II had four variable (V1-V4) and five conserved regions (C1-C5), while group III had six variable (V1-V6) and six conserved regions (C1-C6). Among variable regions, the amino acids of group III and V2 and V3 of group I were the most polymorphic, with H mainly higher than 2.5. Regarding the conserved regions, the C4 of group I, C5 of groups II and III at the C terminus, as well as the C1 of group III at the N terminus were the most conserved regions (Fig 7A). Also, the Omp33-36 variants showed the presence of PLAEAAFL motif at their N terminal region, which was conserved among all the analyzed *Acinetobacter* Omp33-36 porins (S4 File).

### Transmembrane topology of Omp33-36 porin

Transmembrane topology predictions for Omp33-36 variants 2 and 3 showed the same topology scores, 14 β-strand-spanning regions (β1-β14) and seven external loops (L1-L7). However, for the Omp33-36 variants 1 and 4, 12 and 11 β-strand-spanning regions, as well as 6 and 5 external loops were predicted, respectively (Fig 7B).

### PROVEAN analysis

In order to correlate Omp33-36 porin with carbapenem resistance, besides expression level, the Omp33-36 primary sequence also has to be considered. PROVEAN analysis was applied to all four *A*. *baumannii* Omp33-36 variants to reveal whether the change in the primary structure could have an impact on carbapenem resistance. A deleterious effect on variant 1’s protein function resulted from 11 distinct amino acid substitutions and 2 deletions. Omp33-36 variant 2 had 22% deleterious amino acid exchanges, 13 amino acid substitutions, 2 deletions and 1 insertion. The PROVEAN analysis of variant 3 revealed that 28.8% of the mutations had a deleterious effect, 13 amino acid substitutions and 8 deletions. Results for members of variant 4 showed that between 21% and 29.5% of the changes in Omp33-36’s primary structure were deleterious, and the changes were quite diverse (61 distinct mutations). There were 49 amino acid substitutions, 9 deletions and 3 insertions (S1 Table).

## Discussion

Outer membrane porins have been recognized as important factors contributing to both the antimicrobial resistance and virulence of *Acinetobacter* spp. [3,6–9]. Although the role of Omp33-36 porin in *A*. *baumannii* pathogenic potential has been tackled previously [3,6,8,9], data about its gene expression during infection are limited [26,35] and the potential of this porin as a vaccine candidate has been completely neglected.

We investigated the *omp33-36* gene’s transcriptional expression in order to establish its role in reversible resistance to carbapenems caused by inappropriate antibiotic dosing (subinhibitory concentrations of imipenem or meropenem) during different growth phases. Additionally, Omp33-36 porin has been shown to be a virulence factor [8,9], thus we examined the inducibility of the *omp33-36* gene expression during contact with HaCaT keratinocytes. Further, we wanted to determine if there were differences in the transcriptional response of *omp33-36* in one carbapenem-susceptible strain and two carbapenem-resistant strains (with different mechanisms underlying resistance–only production of oxacillinases or in combination with inactivation of the *carO* gene) [22] under the same experimental conditions.

The growth phase dependence of *omp33-36* transcription was undoubtedly demonstrated, since it has been decreased in middle and late exponential phases in all strains. However, the analyzed strains showed different transcriptional responses in the stationary phase. In general, data regarding porin gene expression in *Acinetobacter* spp. are limited and previously it was only observed that the *carO* gene showed significant increase of transcription in the middle exponential phase and subsequent decrease in the following phases, as well as a general trend of decrease of *oprD* gene expression after the early exponential phase [18]. The results obtained in our study indicate that growth phase dependence of the *omp33-36* expression, although showing similar trends, is probably strain-specific, and that correlation with transcription of either the *carO* or the *oprD* genes cannot be taken out. Given that our study analyzed expression at the transcriptional level, the possible variations in the presence of Omp33-36 porin in the outer membrane due to translational or posttranslational regulation should be considered.

Our results suggest the importance of Omp33-36 porin during adaptation to the presence of carbapenems. The most dramatic change in *omp33*-36 expression after treatment with a subinhibitory concentration of carbapenems was detected for the carbapenem-susceptible strain 813. We can assume that this decrease in transcription could further contribute to reduction of Omp33-36 molecules’ number in the outer membrane, limiting entrance of carbapenems and allowing adaptation of the bacteria to new conditions. This indicates how inappropriate carbapenem dosing could lead to reversible resistance, and depicts the role of Omp33-36 during this process. These findings on the hormesis effect of antibiotics, which leads to altered gene expression of resistome genes, are in line with previous studies which have shown that subinhibitory concentrations of antibiotics could lead to amended expression of genetic determinants included in resistance as well as intracellular survival of clinically relevant bacteria [36,37]. Furthermore, the only data that connect the presence of subinhibitory concentrations of antibiotics and the presence of Omp33-36 porin in the membrane are those for tetracycline [35]. Considering that inadequate antibiotic treatment could stimulate the pathogenic properties of clinically relevant bacteria such as *A*. *baumannii*, controlled and proper antibiotic use is imperative for every single infection treatment [38]. The results obtained in this study support previous conclusions, since carbapenem-susceptible strains can become resistant, and MIC values for carbapenem-resistant bacteria can be increased as a result of exposure of bacteria to subinhibitory concentrations of carbapenems.

Since *Acinetobacter* spp. have been recognized as prominent infective agents which cause wound infections [39], their response to contact with keratinocytes is of great importance. The strains we analyzed showed significant increases of *omp33-36* transcription in the presence of HaCaT keratinocytes. Similarly, the increased production of virulence factor OmpA after incubation of *A*. *baumannii* in host airway-like conditions was observed in a previous study [20]. Further, the role of Omp33-36 porin in adhesion and invasion of human lung epithelial cells was established previously [8]. Accordingly, our study revealed that interaction with keratinocytes stimulated the transcription of the *omp33-36* gene encoding virulence factor Omp33-36 in *A*. *baumannii*, indicating that *de novo* synthesis of Omp33-36 porin is important during the initial phases of infection. However, we could not correlate *omp33-36* transcription level and cytotoxicity on HaCaT cells, and we hypothesize that the effect of other virulence factors is more prominent in this interaction than Omp33-36 porin. However, it should be taken into account that the posttranscriptional regulation of *omp33-36* expression, as well as the varying extent of its release in outer membrane vesicles could affect this process [10].

Considering the established importance of Omp33-36 porin in the pathogenicity of *Acinetobacter* spp. [3,6,8,9], the second aim of our study was to classify these outer membrane porins among the genus *Acinetobacter* and species *A*. *baumannii*. Information about Omp33-36 distribution and conservation are needed for assessment of its potential as a vaccine candidate. This analysis was performed according to our previous study in which we revised the classification of another porin, CarO, included in carbapenem resistance[22]. Since the detected average of amino acid polymorphism was over 80%, Omp33-36 could be considered a polymorphic porin. Accordingly, the organization of conserved regions, as well as transmembrane topology, varied significantly among the three different groups. Unlike previously described conserved outer membrane proteins, which showed efficient vaccine characteristics [10–16], only one conserved motif of eight amino acids (PLAEAAFL) was present at the N-terminal region in all three *Acinetobacter* spp. Omp33-36 groups. Since this motif was part of the outside loop L2 of Omp33-36 group II it could be a potential antigen for induction of antibody production against *Acinetobacter* spp. belonging to this group. The investigation on the immunogenicity of this motif and its role in the possible protection of hosts from *Acinetobacter* spp. need to be elucidated in further studies. Although Omp33-36 showed noticeable polymorphism, the majority of these alterations have no influence on its biological function, according to PROVEAN analysis.

## Conclusions

Treatment with subinhibtory concentrations of carbapenems decreased the mRNA levels of *omp33-36*, meaning that inadequate dosing of carbapenem therapy could lead to the development of reversible resistance due to changes in Omp33-36 levels. Contact with keratinocytes increased the expression of *omp33-36*, indicating that the *de novo* synthesis of Omp33-36 is of importance during the initial phases of infection. Bioinformatics analysis revealed the polymorphic structure of Omp33-36 in *Acinetobacter* spp., however, according to PROVEAN analysis, the majority of polymorphism probably does not affect its functionality. We determined a potential antigen region (PLAEAAFL motif) within Omp33-36 from *Acinetobacter* spp. that could be used for vaccine development.

## Supporting information

S1 File Amino acid sequences of Omp33-36 used for bioinformatic analyses. (TXT)

S2 File Nucleotide sequence of the *carO gene* from the *A*. *baumannii* strain 813. (DOC)

S3 File Nucleotide sequence of the *omp33-36 kDa* gene from *A*. *baumannii* strains 813, 1995/12 and 4031. (DOCX)

S4 File Amino acid alignment of *A*. *baumannii* Omp33-36 variant representatives (1–4). (DOCX)

S1 Table Deleterious amino acid changes of *A*. *baumannii* Omp33-36 variants according to PROVEAN analysis. (DOC)
