# Supplementary material for: Acinetobacter spp. porin Omp33-36: Classification and transcriptional response to carbapenems and host cells
Source: PLoS One. 2018 Aug 2;13(8):e0201608. doi: 10.1371/journal.pone.0201608 (PMC6072067; doi:10.1371/journal.pone.0201608)
Supplement: S2 File — (DOC) [file pone.0201608.s002.doc]

Nucleotide sequences of the *carO* gene from isolate *Acinetobacter* *baumannii* 813

ATGAAAGTATTACGTGTTTTAGTGACAACTACAGCTTTACTTGCTGCTGG

TGCCGCAATGGCAGATGAAGCTGTCGTTCATGACAGCTATGCATTTGACC

AAAAACAATTACTT CCTGTAGGGGTGCGTGCTGAGGTAGGTACAACTGGT

TACGGTGGTGCTTTATTATGGCAAGCCAACCCATATGTAGGTTTGGCTCT

GGGTTATAACGGCGGTGACATTTCGTGGACAGATGACGTCTCTGTAAATG

GTACTAAATATGACCTAGATATGGATAACAATAATATTTATCTAAATGCT

GAAATACGTCCTTGGGGCGCTAGTTCTAATCCATGGGCTCAAGGCTTATA

TGTAGCTGCGGGTGCTGCTTATTTAGATAATGATTATGACTTAGCTAAAC

GTATTGGTAATGGCGAAACATTATCAATTGATGGTAAAAACTATCAACAA

GCAGTTGCTGGCCAAGAAGGTGGTGTAAGAGGTAAGATGAATTATGAAAA

TAATATCGCCCCTTATGTAGGTTTTGGTTTTGCACCGAAATTTAATAAAA

ATTGGGGTGTATTTGGTGAAGTAGGTGCTTACTATACAGGTAATCCAAAA

GTTCAGTTAACTCAATATAATCTTGCTCCTGTGAATGGTAACCCAACGTC

TGCTCAAGATGCAGTAGATAAGGAAGAAAATGAAATTCGTAATGACGATA

AATACAAATGGTTACCAGTTGGTAAGGTTGGTGTGAACTTCTACTGG
