# Supplementary material for: Acinetobacter spp. porin Omp33-36: Classification and transcriptional response to carbapenems and host cells
Source: PLoS One. 2018 Aug 2;13(8):e0201608. doi: 10.1371/journal.pone.0201608 (PMC6072067; doi:10.1371/journal.pone.0201608)
Supplement: S3 File — (DOCX) [file pone.0201608.s003.docx]

Nucleotide sequence of the *omp33-36* gene presented in *A. baumannii* strains 813 and 1995/12:

ATGAAAAAACTTGGTTTAGCCACTGCTGTATTATTAGCCATGACCGGTGCTCATGCTTATCAATTTGAAGTTCAAGGTCAATCTGAATATGTTGACACAACTGCAAATGATAAAAACTTCACTGGTGACGTTGCTGGTACATTCTATTTGAAAAATGTTGATACAGCTAAGGGTCCTTTAGCTGAAGCAGCTTTCTTAAACCAAGCTTCTAGCGTGTCTTTAGGTTATAGCTATCAACAATATGACCAAAACAACGTAAACTACCACATTGGTACATACGGTGTTAAAGGTGAGGCATACGTTCCAACTCCTTACCTTCCTGTATATGCTAGCGCAACTTACAACCACACTGATGTTGATGGTAAAAACAACTTCTCTAAAGATGACAACGGTGACCGTTATGCATTAGAAGTTGGTGCTATGTTGTTACCTAACTTCTTAATGACTGTTGGTTATACAAGTGTTGCTAACCAATTCGCTTTAGATAACTTCGGTATCATCGGTAACGGTATCTACTCTGCTGTTAACCAAACTGCTGCTATCCAAAACGACCAAGATGCTGTTACAGCACGTGCTAAATATGTTGGTCCAATCGATGGAACTAACATGGCAATCGGTTTTGAAGCTGCTGGTGCATTCGGTCAAGAAAACCAATACGGTTTAAAAACTGACCTTTACTTAACTCCTAAGTTAAGTGTTGGCGCTACTTTCGTTGGTAACGATGGCGAAGCTGACATCAAAGGTAATGACCTTGGTGAATTCCGCCAAGCTTGGGGTGGTAACGTAAACTACTTCATCACTCCTGCTTTAGCAGTTGGTGCATCTTACATGAAAGCTGACGTTAAAAAGTCTAGCTACGATACACAAACTATCGGCTTAAATGCTAAATTCCGTTTCT

Nucleotide sequence of the *omp33-36* gene presented in *A. baumannii* strain 4031:

ATGAAAAAACTTGGTTTAGCCACTGCTGTATTATTAGCCATGACCGGTGCTCATGCTTATCAATTTGAAGTTCAAGGTCAATCTGAATATGTTGACACAACTGCAAATGATAAAAACTTCACTGGTACTGCTCAAGGTACTTATTACTTCAAAAATGTTGATGCATCTAAAGGTCCTTTAGCTGAAGCAGCTTTCTTAAACCAAGCATCTAACGTTTCTGTTGCTTATAACTATATCAAATATGATGAGAAAGACACTGTAAATGTTGAATCTCATACTTATGGTGTTAAAGGTGAAGCATACCTTCCAACTCCTTACTTACCAGTTTATGCTAGCGCATCTTATAACCACACTATCAATGATTTTAAAGATGGCGTGAGCGATGACAACGGTGACCGTTATGCATTAGAAGCTGGTGCAATGTTGTTACCTAACTTCTTAGTTGCAGTTGGTTATACAAGCGTTGCTGATCAAATTTCTTTAGATGCGTTCGGTGTTAACAAATACGGAATCGCTAAAGCAGTTGGTGAATCAGTTGCTATTGATGAAAAACAAGATGCTGTAACTGCTCGTACTAAATATGTTGGCAACATCGATGGCACTAACATGGCTATTGGTTTTGAAGCATTTGGTGTATTTGCTGAAGACAACGCTTACGGTATGAAAACTGACCTTTTCGTAACTCCTAAGTTAAGTGTTGGTGCATCTTTTGCGGATGTGTCTGCATTTAATTCTGGTTACGACCATGTTTGGGGTGGTCACACTCAATACTTCATCACTCCTGCTGTAGCAGTTGGTGCTGACTTCGTTAAAGCAAATGCGAAAGATGGCAACCCACGTGATACACAAACTATCGGCTTAAATGCTAAATTCCGTTTCT
