# Supplementary material for: Acinetobacter spp. porin Omp33-36: Classification and transcriptional response to carbapenems and host cells
Source: PLoS One. 2018 Aug 2;13(8):e0201608. doi: 10.1371/journal.pone.0201608 (PMC6072067; doi:10.1371/journal.pone.0201608)
Supplement: S4 File — (DOCX) [file pone.0201608.s004.docx]

Amino acid alignment of *A. baumannii* Omp33-36 variants (1-10061; 2-813, 3-WP_000731727.1; 4-WP_049081387.1)

*amino acid N after PLAEAAFL was excluded from conserved motif according to absence of its conservation in alignment done for entire genus *Acinetobacter* (data not shown)
