# Supplementary material for: Acinetobacter spp. porin Omp33-36: Classification and transcriptional response to carbapenems and host cells
Source: PLoS One. 2018 Aug 2;13(8):e0201608. doi: 10.1371/journal.pone.0201608 (PMC6072067; doi:10.1371/journal.pone.0201608)
Supplement: S1 Table — (DOC) [file pone.0201608.s005.doc]

| *A. baumannii* Omp33-36 variant | Type of amino acid variation | Amino acid variation |
| --- | --- | --- |
| 1 | Substitution | H18Q  D32E  N36L  G46_T47delinsIS  Y49_F50delinsFL  F50L  Y81_T93delinsESGDEIATRKNES  Y94F  K123E  V175_V176delinsAL  D224T  S245N |
|  | Insertion | N36_D37insN  N273_A274insYEANL |
|  | Deletion | G246del  G246_D248del |
| 2 | Substitution | K2R |
|  |  | Y20F |
|  |  | Y48F |
|  |  | F50L |
|  |  | A55_S56delinsTA |
|  |  | N71S |
|  |  | V89_H92delinsYHIG |
|  |  | V148_A149delinsMT |
|  |  | D182_K184delinsQNY |
|  |  | A209V |
|  |  | S245_V250delinsGEFRQA |
|  |  | L287F |
|  | Deletion | T282_F293del |
|  |  | F293del |
| 3 | Substitution | M1K |
|  |  | K2S |
|  |  | L4_L6delinsFLF |
|  |  | L6F |
|  |  | T8_G16delinsIFTAVSAFN |
|  |  | Q25_S28delinsGTMY |
|  |  | Y30_V31delinsFT |
|  |  | T33_K38delinsMDGGEV |
|  |  | D54_G58delinsETKNY |
|  |  | P59S |
|  |  | V96_K97delinsIV |
|  |  | V155_K173delinsLSESFDPVQASKYGFITSF |
|  | Deletion | M1_G5del |
|  |  | M1_A7del |
|  |  | P105del |
|  |  | Y106del |
|  |  | T201del |
|  |  | N202del |
|  |  | A289_F293del |
|  |  | F293del |
| 4 | Substitution | M14_G16delinsLGT |
|  |  | M14_H18delinsLAVSG |
|  |  | G16I |
|  |  | G16L |
|  |  | H18G |
|  |  | H18S |
|  |  | Q21K |
|  |  | Q27_Y30delinsTIAL |
|  |  | Q27_V31delinsSAGLT |
|  |  | D54_G58delinsQVKNS |
|  |  | K57Y |
|  |  | A61N |
|  |  | N67S |
|  |  | S73_Y76delinsNAHV |
|  |  | S73_N77delinsKAQYQ |
|  |  | Y76I |
|  |  | K80_E83delinsDYDL |
|  |  | V89_T93delinsSERHR |
|  |  | V89_T93delinsTKDTQ |
|  |  | V89_T93delinsYDETTF |
|  |  | K97_G98delinsGV |
|  |  | G98I |
|  |  | P103S |
|  |  | A113_K123delinsGTIGNINRDSG |
|  |  | A113_S127delinsGSVGRDDSKYD |
|  |  | A113_S127delinsGDVGRNEREIDNTNI |
|  |  | D129_R133delinsFDETV |
|  |  | D129_R133delinsGDTTF |
|  |  | D129_R133delinsSKVTT |
|  |  | G131L |
|  |  | Y152_D157delinsLAGYDN |
|  |  | Y152_L161delinsVKGYDN |
|  |  | Y152_L161delinsVKGYDEKDGK |
|  |  | D186V |
|  |  | D186_V188delinsADP |
|  |  | D186_V188delinsVDP |
|  |  | G196_G200delinsTTLSN |
|  |  | N202_F207delinsGNDINL |
|  |  | S245_Y257delinsINNSDVFTIRAKK |
|  |  | S245_Y257delinsPYDSNEFGVSARK |
|  |  | I259_A262delinsLNQQ |
|  |  | P261E |
|  |  | V265_A274delinsLEGRVGFGEI |
|  |  | G266_A274delinsEGSIDFAEE |
|  |  | R280_I285delinsYNKF |
|  |  | T284I |
|  |  | L287G |
|  |  | N288A |
|  |  | N288R |
|  | Insertion | H18_A19insAAN |
|  |  | H18_A19insTAN |
|  |  | H18_A19insTAAH |
|  | Deletion | P105del  Y106del  G125_S127del  L161_A163del  F164_Q185del  G200del  T201del  K275_D281del  T284_F293del |
|  |  |  |
